# Identification of Genes Related to 5-Fluorouracil Based Chemotherapy for Colorectal Cancer

**DOI:** 10.3389/fimmu.2022.887048

**Published:** 2022-06-17

**Authors:** Xingxing Huang, Kun Ke, Weiwei Jin, Qianru Zhu, Qicong Zhu, Ruyi Mei, Ruonan Zhang, Shuxian Yu, Lan Shou, Xueni Sun, Jiao Feng, Ting Duan, Yiping Mou, Tian Xie, Qibiao Wu, Xinbing Sui

**Affiliations:** ^1^ State Key Laboratory of Quality Research in Chinese Medicines, Faculty of Chinese Medicine, Macau University of Science and Technology, Macau, Macau SAR, China; ^2^ School of Pharmacy and Department of Medical Oncology, The Affiliated Hospital of Hangzhou Normal University, Hangzhou Normal University, Hangzhou, China; ^3^ Key Laboratory of Elemene Class Anti-Cancer Chinese Medicines, Engineering Laboratory of Development and Application of Traditional Chinese Medicines, Collaborative Innovation Center of Traditional Chinese Medicines of Zhejiang Province, Hangzhou Normal University, Hangzhou, China; ^4^ Department of Gastrointestinal-Pancreatic Surgery, Zhejiang Provincial People’s Hospital, People’s Hospital of Hangzhou Medical College, Hangzhou, China; ^5^ Guangdong-Hong Kong-Macau Joint Laboratory for Contaminants Exposure and Health, Guangzhou, China

**Keywords:** immune-related genes, tumor microenvironment, colorectal cancer, 5-FU resistance, prognosis

## Abstract

**Background:**

Colorectal cancer (CRC) is one of the most common malignancies and its incidence and mortality are increasing yearly. 5-Fluorouracil (5-FU) has long been used as a standard first-line treatment for CRC patients. Although 5-FU-based chemotherapy is effective for advanced CRC, the consequent resistance remains a key problem and causes the poor prognosis of CRC patients. Thus, there is an urgent need to identify new biomarkers to predict the response to 5-FU-based chemotherapy.

**Methods:**

CRC samples were retrieved from Gene Expression Omnibus (GEO) and The Cancer Genome Atlas (TCGA). The immune-related genes were retrieved from the ImmPort database. Single-cell sequencing results from colorectal cancer were obtained by the ArrayExpress database. 5-FU resistance-related genes were filtered and validated by R packages. ESTIMATE algorithms were used to assess the tumor microenvironment (TME). KEGG and GO analysis were performed to explore the biological signaling pathway for resistant-response patients and sensitive-response patients in the tumor microenvironment. pRRophetic algorithms were used to predict 5-FU sensitivity. GSEA and GSVA analysis was performed to excavate the biological signaling pathway of the RBP7 gene.

**Results:**

Nine immune-related genes were identified to be associated with 5-FU resistance and poor disease-free survival (DFS) of CRC patients and the signature of these genes was developed in a DFS-prognostic model. Four immune-related genes were determined to be associated with 5-FU resistance and overall survival (OS) of CRC patients. The signature of these genes was developed an OS-prognostic model. ESTIMATE scores showed a significant difference between 5-FU resistant and 5-FU sensitive CRC patients. Resistant-response patients and sensitive-response patients to 5-FU based chemotherapy showed different GO and KEGG enrichment on the tumor microenvironment. RBP7, as a tumor immune microenvironment (TIME) related gene, was found to have the potential of predicting chemotherapy resistance and poor prognosis of CRC patients. GSEA analysis showed multiple signaling differences between the high and low expression of RBP7 in CRC patients. Hypoxia and TNFα signaling *via* NFκB gene sets were significantly different between chemotherapy resistant (RBP7^High^) and chemotherapy sensitive (RBP7^Low^) patients. Single-cell RNA-seq suggested RBP7 was centrally distributed in endothelial stalk cells, endothelial tip cells, and myeloid cells.

**Conclusions:**

Immune-related genes will hopefully be potential prognostic biomarkers to predict chemotherapy resistance for CRC. RBP7 may function as a tumor microenvironment regulator to induce 5-FU resistance, thereby affecting the prognosis of CRC patients.

## Background

Colorectal cancer (CRC) is a common malignant tumor with a high incidence and one of the leading causes of cancer-related mortality worldwide. The five-year survival rates of stage I or II diseases are 91 percent and 82 percent, respectively, but the data for patients with metastatic disease is only 12% (1). Surgery is strongly recommended for the early and local advanced CRC (2, 3). According to the stage defined of American Joint Committee on Cancer, patients with stage I and II disease have a 30% chance of recurrence after surgical performance within five years, whereas the chance for patients with stage III disease has up to a 50-60% (4–6). Thus, 5-fluorouracil (5-FU) based regimens followed by surgery have become the standard treatment and significantly reduce the risk of recurrence for patients with stage III and high-risk stage II CRC (1). Most patients can benefit from chemotherapy, while others do not, and may suffer ineffective chemotherapy for several cycles and even die from side effects (7, 8). The reason why these patients show a nonresponse tochemotherapy is the resistance to drugs.

The resistant patterns of cancers cells to 5-FU-based therapy include primary (innate) drug resistance and secondary (acquired) drug resistance. Both primary and secondary drug resistance involves multiple molecular mechanisms. A high level of thymidylate synthase (TS) was linked to decreased sensitivity to 5-FU-based therapy (9, 10). High dihydropyridine dehydrogenase (DPD) activity might be correlated to the drug resistance by reducing the toxicity and catabolism of 5 FU (11, 12). Also, thymidine phosphorylase (TP) is also involved in the resistance of 5-FU treatment (13). Due to the limitations of clinicopathologic variables for prognostic prediction, the stratification of chemotherapy response based on biological characteristics is essential for identifying the treatment sensitivity of CRC patients. Although some studies have reported genes and signal pathways related to 5-FU resistance, novel biomarkers to predict response to 5-FU-based chemotherapy are urgently needed.

The tumor microenvironment (TME) is composed of various infiltrating cells, including immune cells, inflammatory cells, vascular endothelial cells, and their associated mediators in and around the tumor (14). The cellular components within the TME play an important role in oncogenesis, tumor metastasis, and drug response (15). The tumor immune microenvironment (TIME) modulates cancer development by infiltrating immune cells, such as T lymphocytes, B lymphocytes, and natural killer (NK) cells (16). T cells are the most characterized immune cells in the TIME of solid tumors (15, 16). The TIME has a prime role in mediating cytotoxic drug response and resistance (17). The TIME baseline of CRC patients may promote immune evasion through low antigenicity, absence of immune effectors, or immunosuppression, which may facilitate primary resistance to chemotherapy (18).

The tumor immune microenvironment is an important part of the tumor microenvironment. In recent years, it has been found that the TIME is related to cancer drug resistance. However, the relationship between 5-FU resistance and immune-related genes remains unclear. In this study, we identified immune-related genes to 5-FU resistance in the tumor microenvironment and found that RBP7 has the potential to predict chemotherapy resistance and poor prognosis of CRC patients, which will hopefully provide a biomarker for 5-FU resistance and prognosis for CRC patients.

## Materials and Methods

### Data Collection

**Figure 1:**
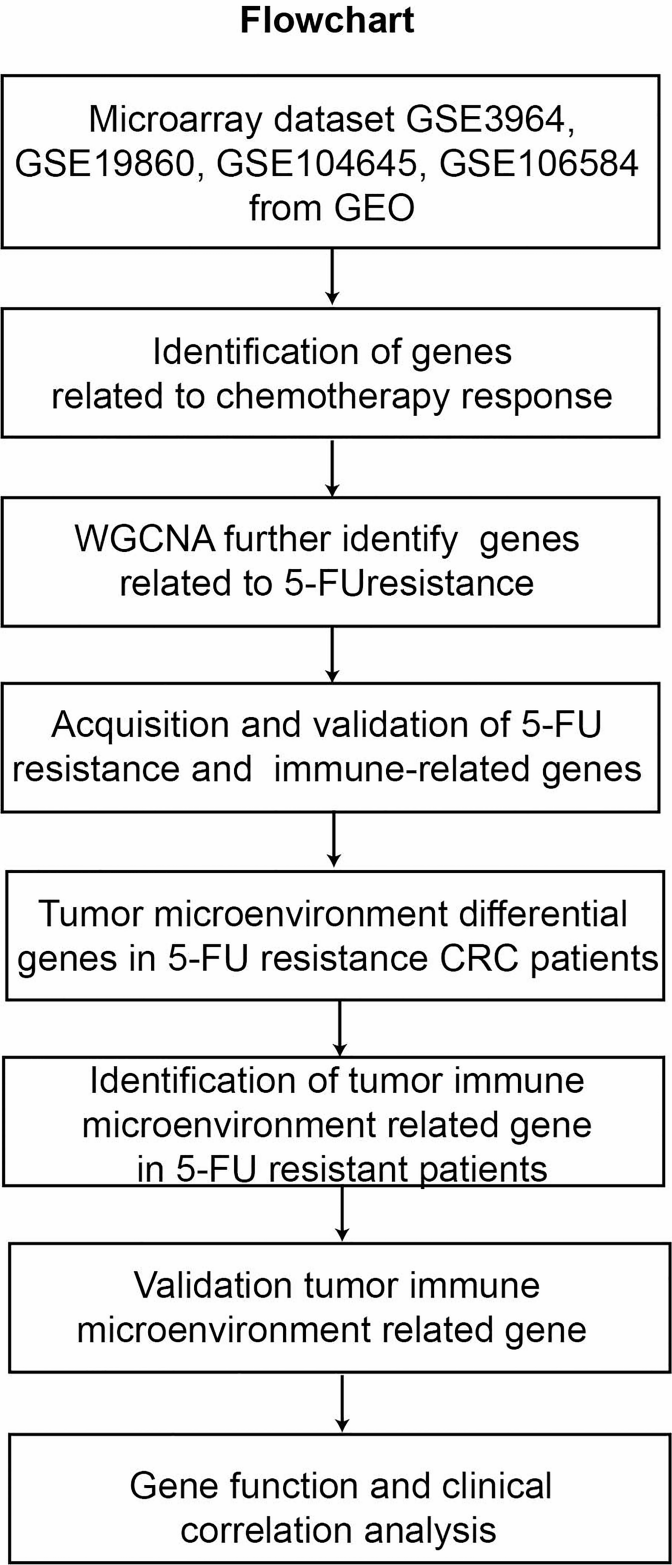
The workflow for analyzing the tumor immune microenvironment related gene to 5-FU resistance in CRC.

A flowchart of this study is presented in **Figure 1**. Gene expression profiles of Datasets (GSE3964, GSE19860, GSE104645, GSE106584, GSE69657) were downloaded from the Gene Expression Omnibus (GEO) database (https://www.ncbi.nlm.nih.gov/geo/query/acc.cgi). A total of 230 CRC samples were retrieved for subsequent analysis from the five datasets. The first four datasets contained expression profiling of 200 clinical samples collected from CRC patients before the exposure to 5-FU-based chemotherapy and the last one contained expression profiling of 30 clinical samples collected from CRC patients after the exposure to 5-FU-based chemotherapy. Analysis of gene expression profiles between chemotherapy-resistant patients and chemotherapy-sensitive patients in datasets GSE3964, GSE19860, GSE104645, and GSE106584 were used to identify biomarkers associated with innate drug responses. Gene expression profiles of dataset GSE69657 were used to explore the tumor microenvironment expression patterns of the patients who received 5-FU-based chemotherapy. Another gene expression profile (FPKM) of 584 CRC patients (426 COAD, 158 READ) was downloaded from UCSC (https://gdc.xenahubs.net/download/TCGA-COAD.htseq_fpkm.tsv.gz; https://gdc.xenahubs.net/download/TCGA-READ.htseq_fpkm.tsv.gz). Samples with a follow-up time of fewer than 30 days were excluded.

### Primary Filtering of Differentially Expressed Genes

Included in our study were 46 CRC patients of GSE3964, 29 CRC patients of GSE19860, 54 CRC patients of GSE104645, and 71 CRC patients of GSE106584. Differentially expressed genes (DEGs) in datasets GSE3964, GSE19860, GSE104645, and GSE106584 were analyzed by R packages, respectively. Genes with p < 0.05 was considered a significantly differentially expressed gene between the chemotherapy-resistant and chemotherapy-sensitive patients.

### Secondary Filtering of Differentially Expressed Genes

The DEGs of datasets GSE3964, GSE19860, GSE104645, and GSE106584 were separately constructed to gene co-expression networks using the WGCNA package in R to seek the modules of highly associated genes among samples for relating modules to external sample traits (19). A weighted adjacency was constructed by calculating Pearson correlations of all gene pairs and a soft power value was selected to construct a standard scale-free network in each dataset. The similarity matrix completed by Pearson correlation of all gene pairs was transformed into topological overlap matrix (TOM) and corresponding dissimilarity (1-TOM). Similar gene expression was classified into different gene co-expression modules using a hierarchical clustering dendrogram of the 1-TOM matrix. Module-resistant associations were calculated to locate functional modules in the co-expression network. Modules with high correlation coefficients (the absolute value of correlation coefficient ≥0.4, P<0.05) were considered to be related to resistance and were extracted for further analysis.

### Extraction of Immune Related Genes

The immune-related genes (IRGs) list was retrieved from the ImmPort database (https://www.immport.org/shared/genelists). Overlapping immune-related genes, from the results of WGCNA and the IRGs, were selected for further analysis.

### Validation of 5-FU Resistance and Immune Both Related Genes

Both related genes from the GSE106584 were verified on survival data from itself. The genes screened from the other GEO datasets were validated on survival data from the TCGA cohort. Univariate Cox regression analysis was used to filter all candidate genes and multivariate Cox regression analysis was used to filter the genes from univariate Cox regression analysis. The meaning genes from multivariate Cox regression analysis were selected and compared to the expression of these genes of 5-FU resistant and 5-FU sensitive patients in each dataset.

### Tumor Microenvironment Differentially Expressed Genes in 5-FU Resistance CRC Patients

The ESTIMATE algorithm was used to determine the scores of CRC patients in GSE69657 to explore the role of the tumor microenvironment in 5-FU resistance. We compared the differentially expressed genes of the high-score and low-score groups to acquire 5-FU resistance-related genes in the tumor microenvironment.

### Prediction of Response to 5-FU

The R package of pRRophetic was used to predict IC50 of 5-FU in GSE19860. IC50 implies the efficiency of a substance in restraining certain biological or biochemical functions. “cgp2016” was selected to be the predicted library (20). The quartile of RBP7 expression as the cut-off, patients were divided into RBP7-high expressed and RBP7-low expressed groups.

### GO and KEGG Functional Enrichment Analysis

Gene ontology (GO) and Kyoto Encyclopedia of Genes and Genomes (KEGG) enrichment analyses for genes in the tumor microenvironment differential genes of 5-FU resistance were performed using an R package “clusterProfiler”. GO annotation was based on three categories including, biological processes (BP), cellular compartments (CC), and molecular functions (MF). Terms in GO and KEGG with adj.P.value < 0.05 were considered significantly enriched and were visualized by R package “enrichplot” and “ggplot2”.

### Clinical Analysis

Excluding incomplete information samples, there were 401 of 584 CRC patients (286 COAD, 115 READ) to be explored the RBP7 clinical meaning in the TCGA cohort. The median of RBP7 expression as the cut-off, patients were divided into RBP7-high expressed and RBP7-low expressed groups. Chi-square or Fisher’s exact tests were used to compare the differences in clinical parameters between RBP7-high expressed and RBP7-low expressed groups.

### Gene Set Enrichment Analysis (GSEA)

To identify signaling pathways that are differentially activated in 5-FU resistance CRC with different expression RBP7, we selected an ordered list of genes through the limma R package and conducted Gene Set Enrichment Analysis (GSEA) with adjusted p < 0.05 using the clusterfiler R package in the GSE19860 dataset, in which BBP7 expression was statistically different between the 5-Fu sensitive and the resistant groups.

### Gene Set Variation Analysis (GSVA)

To identify downstream signaling pathways that are differentially activated in 5-FU resistance CRC with different expression RBP7, we performed GSVA with adjusted p < 0.05 to explore correlated pathways of RBP7 in the GSE19860. Hallmark gene sets “h.all.v7.5.1.symbols.gmt” were downloaded from Molecular Signatures Database (https://software.broadinstitute.org/gsea/downloads.jsp).

### Single-Cell RNA-Seq (scRNA-Seq) Analysis

The cell plots of single-cell sequencing of colorectal tumors and adjacent non-tumor colon tissue were downloaded from ArrayExpress databases (https://www.ebi.ac.uk/gxa/sc/home). We searched for colorectal cancer, obtained the result of single-cell sequencing for colorectal tumors and adjacent non-malignant colon tissue, drew pictures online, and downloaded. Drawing path: plot type: UMAP, plot options: n_neibors:100, color plot by: ontology labels, gene name: RBP7.

### Cell Culture

Lovo and Lovo/5-FU cells (induced by increasing continuous exposure concentration of parental cells Lovo) were cultured in DMEM/F-12 medium (BasalMedia) containing 10% fetal calf serum (BI), and 100 U/ml each of penicillin and strepcomycin (BI) at 37°C with 5% CO2. The maintenance concentration of Lovo/5-FU cells was 0.01mg/mL.

### Establishment of 5-FU Resistant Cells

To establish 5-FU resistant cells, Lovo cells were induced by increasing continuous exposure concentration. Lovo cells cells were treated in a culture medium containing 0.1μg/mL 5-FU. When the cell survival rate was greater than 90% and the cell growth could be maintained, the dose was increased by 1.5 times, and repeated until the cells could grow stably in the culture medium with 5-FU concentration of 0.01mg/ml. The 5-FU resistant Lovo cells were obtained by continuous exposure to gradually increased concentrations of 5-FU for eight months.

### Western Blot Analysis

Proteins were separated by 10% SDS-PAGE and then transferred onto a nitrocellulose membrane. The following primary antibodies were used: anti-GAPDH (Cell Signaling Technology) and anti-RBP7 (ABclonal Technology). The following secondary antibody was used: goat anti-rabbit IgG-HRP antibody. The proteins were visualized using an ECL detection kit (FDBIO).

### Plasmids

Full-length RBP7 DNA was amplified by PCR with the primer 5’-CTTTGCCACTCGTAAAATAGCCA-3’ and 5’-CGTGTGGATGGTAAAAGAATCCC-3’ (Tsingke Biotechnology, China). Full-length GAPDH DNA was amplified by PCR with the primer 5’- GGAGCGAGATCCCTCCAAAAT-3’ and 5’-GGCTGTTGTCATACTTCTCATGG-3’ (Tsingke Biotechnology).

### Statistical Analysis

All statistical analyses were performed using Prism (version 8) and R (version 4.1.2). The proportional composition of the two variables was compared using the Chi-square or Fisher’s exact tests. The comparison of RBP7 expression and the ESTIMATE scores between the two datasets were performed using the Mann-Whitney U tests. Predicted 5-FU sensitivity difference between groups was tested by unpaired t test.The median value was set as the cut-off. Survival analysis, WGCNA, ESTIMATE algorithm, and the COX regression analysis were carried out by R version 4.1.2 and corresponding packages. Statistical significance was set at P.value <0.05 or adj.P.value < 0.05.

## Results

### Differentially Expressed Genes (DEGs) From GEO Datasets

**Figure 2:**
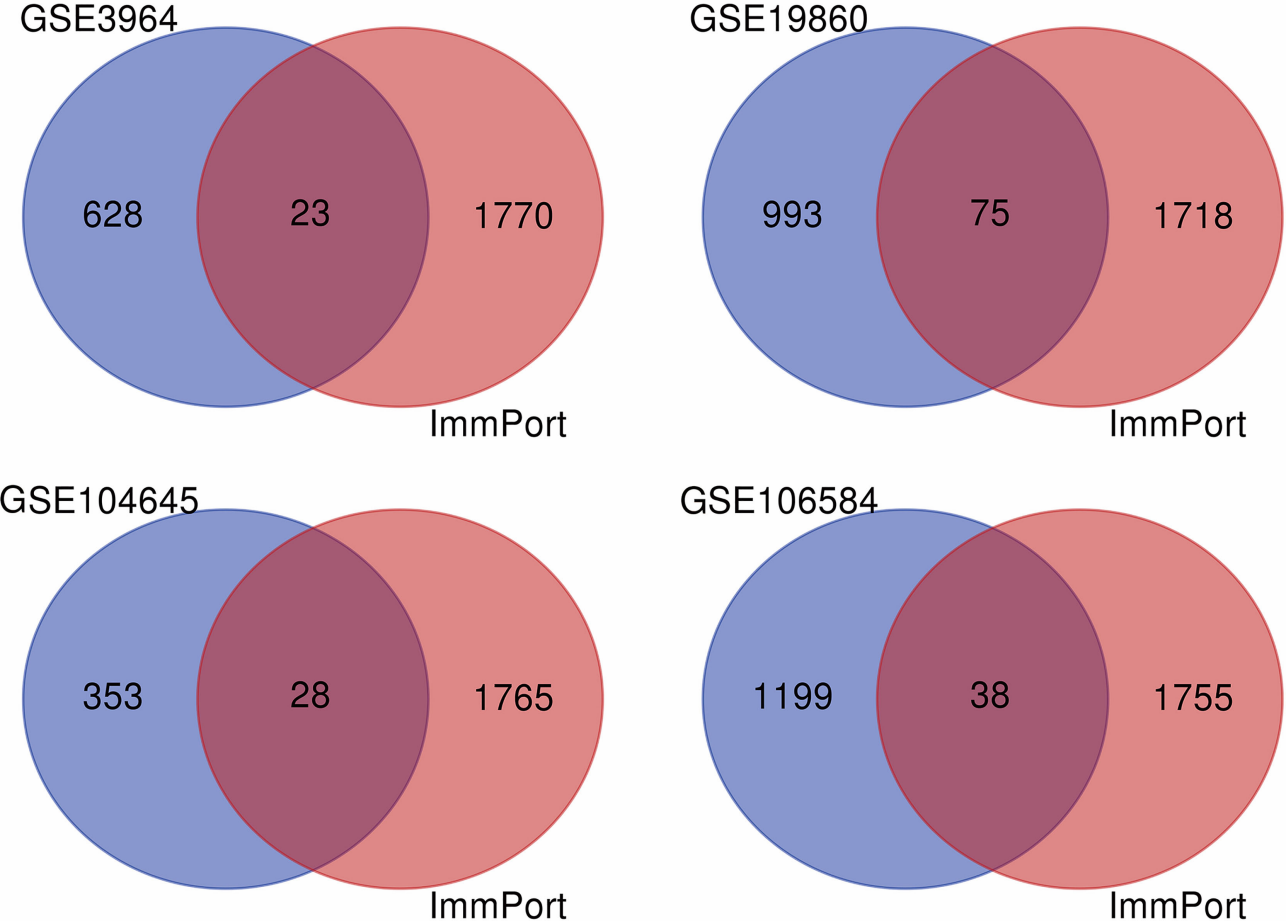
The VENN diagram for the intersection of DEGs and IRGs among GEO database and ImmPort database. The blue part was DEGs in GEO datasets, the red part was immune genes in ImmPort database, and the intersection part of the two was immune-related genes of 5-FU chemotherapy sensitivity.

Firstly, datasets GSE3964, GSE19860, GSE104645, and GSE106584 all contained chemotherapy-sensitive and chemotherapy-resistant samples. To explore the differentially expressed genes related to chemotherapy response, we used the limma package for differential analysis. There were 1,068 DEGs in the GSE19860 mRNA profile, 651 DEGs in the GSE3964 mRNA profile, 1,392 DEGs in the GSE 104645 mRNA profile, and 1,237 DEGs in the GSE 106584 mRNA profile (**Figure 2**).

### Weighted Gene Co-Expression Network Analysis and 5-FU Resistance Related Genes Further Identification

**Figure 3:**
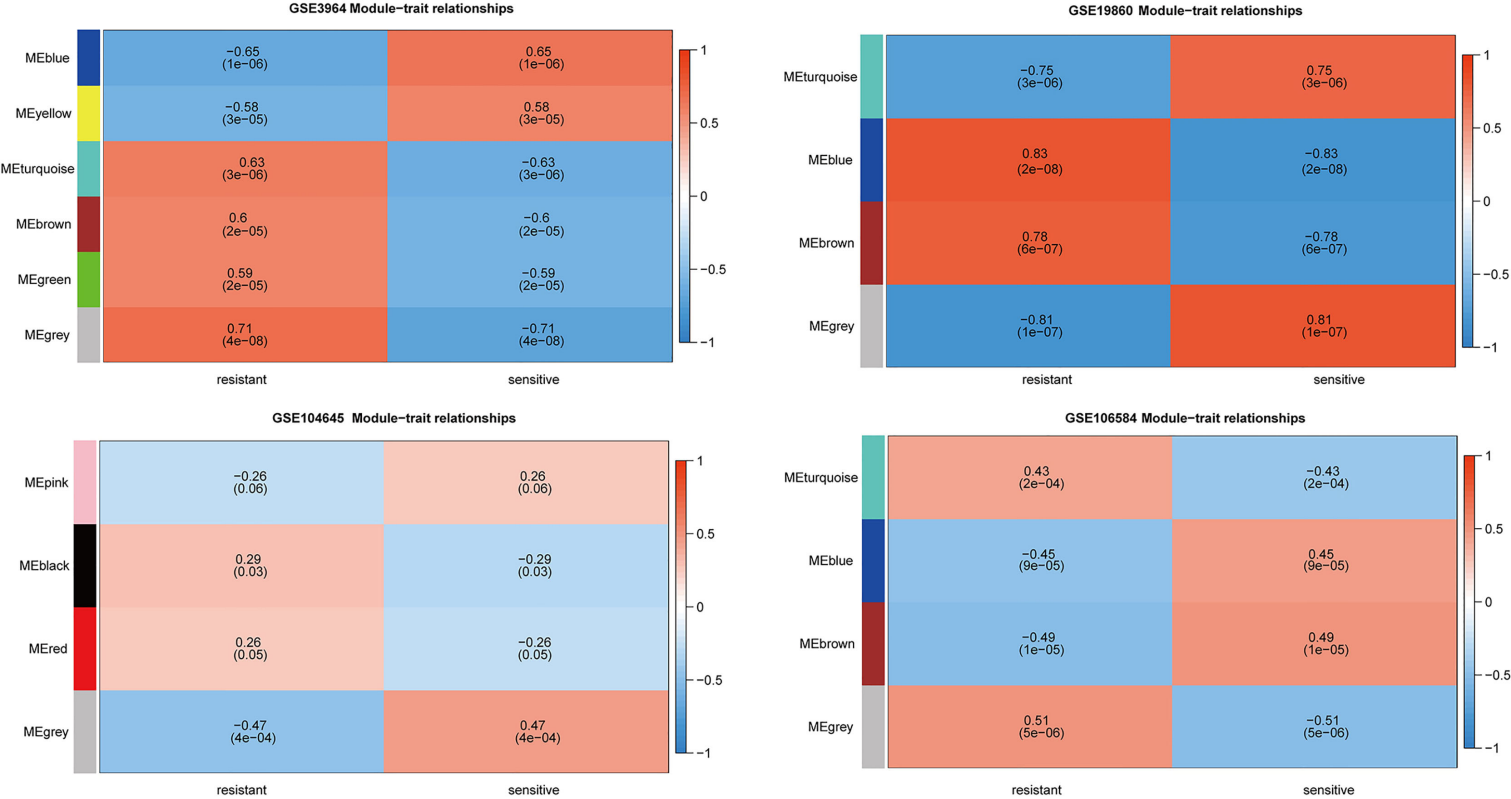
Relationships between the module and clinical traits for four GEO datasets. Each row represents a color module and column corresponds to 5-FU resistant or 5-FU sensitive. Each cell contains the corresponding correlation and p-value.

To further explore the functional clusters related to 5-FU based chemotherapy resistance, the weighted gene co-expression network was constructed from the DEGs sets which filter from GSE3964, GSE19860, GSE104645, and GSE106584. The soft-thresholding power in WGCNA was determined based on a scale-free R2 (R2 = 0.9). Hub modules were identified based on the average linkage hierarchical clustering and the soft-thresholding power. To evaluate the link between modules and clinical traits (5-FU resistant and 5-FU sensitive), the heatmap of the module-trait relationship was plotted (**Figure 3**). All modules showed a high correlation with 5-FU resistance of CRC in GSE3964 and contained 651 5-FU resistance-related genes. All modules showed a high correlation with 5-FU resistance of CRC in GSE19680 and contained 1,068 5-FU resistance-related genes. Only one module showed a high correlation with 5-FU resistance in GSE104645 and contained 381 5-FU resistance-related genes. All modules showed a high correlation with 5-FU resistance of CRC in GSE106584 and contained 1,237 5-FU resistance-related genes.

### Identification of 5-FU Resistance and Immune Both Related Genes

After differentially expressed genes analysis and further screening by WGCNA, we identified gene clusters associated with 5-FU resistance. We obtained 2,483 immune-related genes from the Immport database. The genes related to 5-FU resistance, screened out from GEO datasets, were intersected with immune-related genes to obtain immune-related genes to 5-FU resistance (**Figure 2**). There were 23 IRGs to 5-FU resistance for CRC patients in GSE3964. There were 75 IRGs to 5-FU resistance for CRC patients in GSE19860. There were 28 IRGs to 5-FU resistance for CRC patients in GSE104645. There were 39 IRGs to 5-FU resistance for CRC patients in GSE106584. HLA-DQA1 and MX2 were both in GSE104645 and GSE19860. TUBB3 was also found both in GSE19860 and GSE3964. One research suggested that patients with high TUBB3 had a statistically significant poorer OS when undergoing docetaxel-based versus 5-FU/LV chemotherapeutic (21).

### Verification of 5-FU Resistance and Immune Both Related Genes in GEO and TCGA Cohort

The IRGs from GSE106584 were verified on its survival data and the IRGs from the other three datasets were verified on survival data in the TCGA cohort. In GSE106584, univariate Cox regression analysis suggested that 15 genes were related to DFS, and 9 genes mean to OS as well. Meanwhile, in the TCGA cohort, 19 genes were mean to OS and 9 genes were correlated to DFS, but none of the genes correlated to OS in the GSE106584 resulted from the multivariate Cox regression. In The TCGA cohort, 4 genes were associated with OS by the multivariate Cox regression analysis. Finally, we found 13 IRGs linked to 5-FU resistance.

### Development of the Prognostic Gene Signature

**Figure 4:**
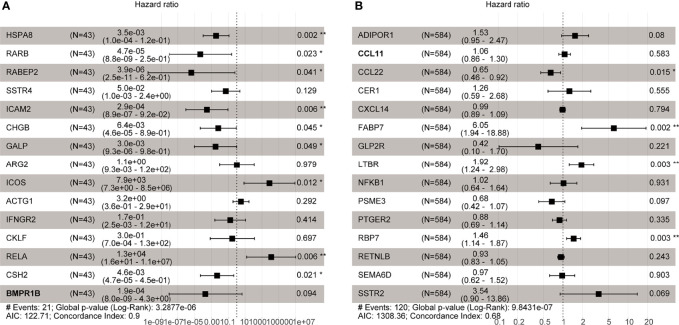
Multivariate Cox regression analysis in GSE106584 and TCGA cohort. **(A)** There were 9 genes related to DFS in GSE106584. **(B)** There were 4 genes related to OS in TCGA cohort *p-value < 0.05; **p-value < 0.001.

**Figure 5:**
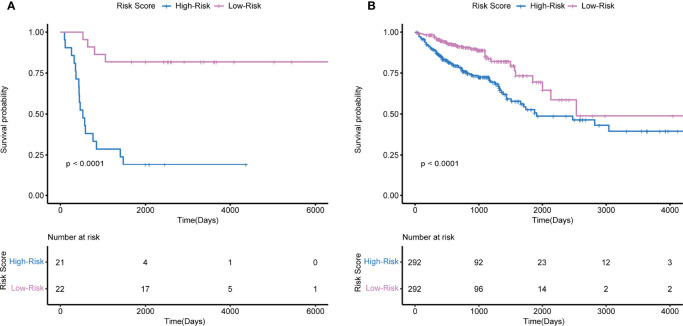
Kaplan-Meier survival based on the integrated classifier in the GSE106584 and TCGA cohort. **(A)** KM curve of nine-genes DFS-prognostic signature in GSE106584. **(B)** KM curve of four-genes OS-prognostic signature in TCGA cohort.

Multivariate Cox regression analysis was used to develop immune-related prognostic gene signatures. After obtaining the coefficients of each gene, we calculated the risk score of each CRC patient with the computational equation. In GSE106584, the DFS-prognostic model included nine genes: HSPA8, RARB, RABEP2, ICAM2, CHGB, GALP, ICOS, RELA, and CSH2 (**Figure 4A**). In the TCGA cohort, the OS-prognostic model contained four genes: CCL22, FABP7, LTBR, and RBP7 (**Figure 4B**). Risk scores were constructed with the regression coefficients from these models and thresholds were chosen manually at the median. In the DFS-prognostic model, high-risk patients had significantly worse DFS (*P* <0.0001) (**Figure 5A**). In the OS-prognostic model, high-risk patients had statistically notable worse OS (*P* <0.0001) (**Figure 5B**).


$$Risk\;score\;=\;\sum_{n=1}^{\infty }(coefficient_{n}{\text{expgene}}_{n})$$

### Differentially Expressed Genes of Tumor Microenvironment to 5-FU Resistance in CRC Patients

**Figure 6:**
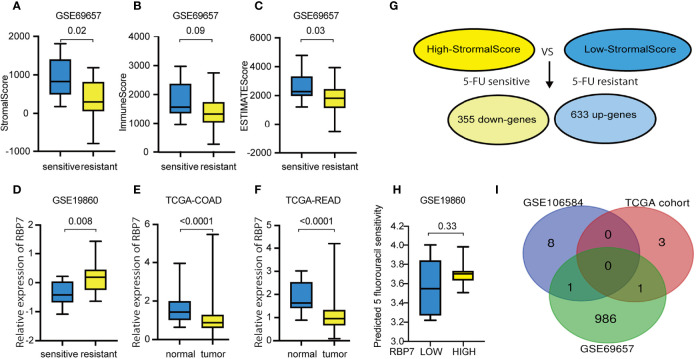
Tumor microenvironment score in CRC with different chemotherapy responses and tumor microenvironment related genes to 5-FU resistance in GSE69657. **(A)** 5-FU resistant patients showed statistically significant lower StromalScore. **(B)** 5-FU resistant patients showed lower ImmuneScore, but not statistically significant. **(C)** 5-FU resistant patients showed statistically significant lower ESTIMATEScore. **(D)** RBP7 was up-regulated in CRC patients with 5-FU resistance. **(E)** RBP7 was down-regulated in COAD compared with normal tissue. **(F)** RBP7 was down-regulated in READ compared with normal tissue. **(G)** Compared high StromalScore group with low StromalScore group, there were 355 down-expressed genes and 633 up-expressed genes in GSE69657. **(H)** The predicted 5 fluorouracil sensitivity in RBP7 subgroups. **(I)** Intersecting previously screened immune-related drug resistance genes with tumor microenvironment related genes and obtained two immune-related genes to 5-FU resistance genes in tumor microenvironment.

The ESTIMATE algorithm was used to determine the scores of each sample in dataset GSE69657 by R software. The scores showed a remarkable difference between the 5-FU resistant and the 5-FU sensitive groups. 5-FU resistant patients showed lower scores and were statistically significant in StromalScore and ESTIMATEScore but not in ImmuneScore (**Figures 6A–C**). Furthermore, we compared the differentially expressed genes between high StromalScore and low StromalScore groups to obtain tumor microenvironment-related genes. We found two gene sets which contained 988 genes: 355 down-expressed genes and 633 up-expressed genes (**Figure 6G**).

### Functional and Pathway Enrichment Analysis

**Figure 7:**
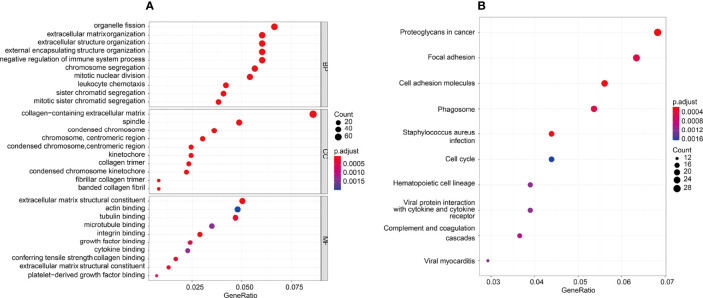
GO and KEGG enrichment analysis was performed in the tumor microenvironment to 5-FU resistance. **(A)** GO enrichment analysis, there were mostly enriched in organelle fission, extracellular matrix organization and extracellular structure organization on BP enrichment. There were mainly involved in collagen−containing extracellular matrix and spindle on CC enrichment. There were mainly enriched in extracellular matrix, tubulin binding and actin binding on MF enrichment. **(B)** KEGG enrichment, top10 pathways: Proteoglycans in cancer, Focal adhesion, Cell adhesion molecules, Staphylococcus aureus infection, Phagosome, Complement and coagulation cascades, Hematopoietic cell lineage, Viral myocarditis, Viral protein interaction with cytokine and cytokine receptor, and Cell cycle.

The potential function of the tumor microenvironment-related genes to 5-FU resistance was performed by GO and KEGG enrichment analysis. For BP enrichment, the genes most enriched in organelle fission, extracellular matrix organization, and extracellular structure organization are shown in **Figure 7A**. For CC enrichment, the genes most enriched in the collagen-containing extracellular matrix and spindle are shown in **Figure 7A**. For MF enrichment, the genes most enriched in the extracellular matrix, tubulin binding, and actin-binding are shown in **Figure 7A**. For KEGG enrichment, large portion of the genes concentrated on proteoglycans in cancer, focal adhesion, cell adhesion molecules, staphylococcus aureus infection, phagosome, complement and coagulation cascades, hematopoietic cell lineage, viral myocarditis, viral protein interaction with cytokine, cytokine receptor, and cell cycle are shown in **Figure 7B**).

### Identification of Tumor Immune Microenvironment Related Gene of 5-FU Resistance

We obtained immune-related genes of 5-FU resistance genes in the tumor microenvironment by intersecting previously screened, immune-related drug resistance genes with tumor microenvironment-related genes (**Figure 6I**). We gained two overlapping genes RARB and RBP7 and further verified the expression of these two genes in 5-FU-sensitive and -resistant patients in the other three GEO datasets. RBP7 was up-regulated in patients with 5-FU- resistance and was statistically significant in GSE19860 (**Figure 6D**). However, RBP7 was down-regulated in CRC tissue compared with normal tissue in the TCGA cohort (**Figures 6E, F** **)**. In addition, in GSE19860, the IC50 of 5-FU predicted by "pRRophetic" in patients with high expression of RBP7 was higher than patients with low RBP7 expression, but the results were not statistically significant(p-value=0.33)(**Figures 6H**). We defined the RBP7 as a tumor immune microenvironment-related gene for CRC to 5-FU resistance.

### Clinical Analysis

We selected the median as the cut-off to divide the groups into the RBP7-high expressed and RBP7-low expressed groups. We analyzed the relationship between the expression of RBP7 and the clinical characteristics of CRC patients and the results showed that the expression of RBP7 was closely correlated with T stage, but not with age, N stage, M stage, or mismatch repair gene deletion (**Table 1**). Although RBP7 was not associated with lymphatic invasion, it did mean for the number of lymph nodes. Also, RBP7 expression was linked to survival status.

**Table 1:** Clinical feature of colorectal cancer patients of RBP7 expression (TCGA cohorts).

clinical variables	levels	Low-RBP7(n=200)	High-RBP7(n=200)	P value
Tumor site	COAD	142	143	0.91
	READ	58	57	
Age	<65	87	82	0.61
	≥65	113	118	
T	T1	9	4	<0.0001****
	T2	52	18	
	T3	125	147	
	T4	14	32	
	Tis	1	0	
N	N0	119	101	0.13
	N1	49	53	
	N2	32	46	
M	M0	160	149	0.38
	M1	24	33	
	MX	16	18	
lymph node	<12	8	21	0.02*
	≥12	191	176	
	NA	1	3	
lymphatic invasion	Yes	86	84	0.84
	No	114	116	
MMR	dMMR	167	174	0.32
	pMMR	33	26	
Survival status	Alive	179	163	0.02*
	Death	21	37	
OS	1 year	163	156	0.78
	3 year	99	85	
	5 yuer	10	11	

### GSEA Analysis

**Figure 8:**
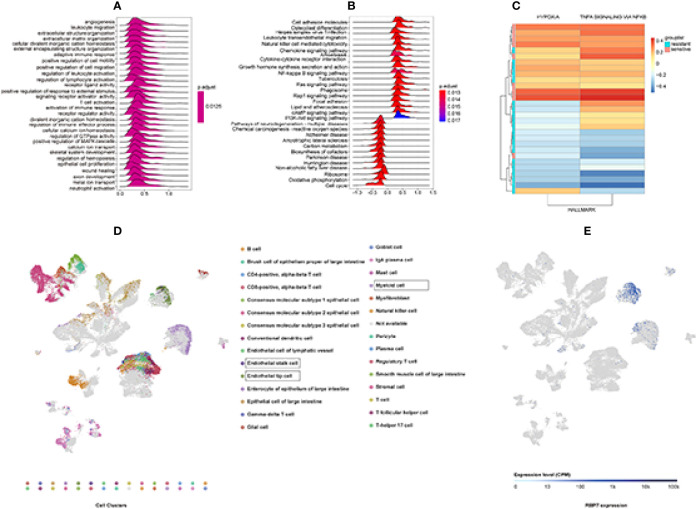
GSEA and GSVA analysis to explore RBP7 function enrichment based on GSE19860 and Single-Cell RNA-seq Analysis results from ArrayExpress databases. **(A)** The top 30 significant GO terms. **(B)** The top 30 significant KEGG pathways. **(C)** Hypoxia and TNFα signaling *via* NFκB gene sets were significantly different between chemotherapy resistant (RBP7^High^) and chemotherapy sensitive (RBP7^Low^) patients in GSE19860. **(D)** 30 clusters of the Single-Cell RNA-seq Analysis. **(E)** Distribution of RBP7 in colorectal cancer patients.

GSEA analysis showed 36 significant KEGG pathways were associated with RBP7 expression. The top 10 percent of pathways included Herpes simplex virus 1 infection, PI3K-Akt signaling pathway, and cytokine-cytokine receptor interaction. GO enrichment for 593 gene sets was performed. 480 gene sets were enriched in the BP process, 65 gene sets accessed in the CC enrichment, and only 48 gene sets were acquired in the MF enrichment. The top 30 significant GO term and KEGG pathways are shown in **Figures 8A, B**.

### GSVA Analysis

In order to further obtain information related to the function of RBP7 gene, we selected GSE19860 dataset for GSVA analysis to obtain enrichment of downstream pathways related to RBP7. We found that two pathways gene sets were significantly altered in chemotherapy-sensitive (RBP7^Low^) and chemotherapy-resistant (RBP7^High^) patients, and those were Hypoxia and TNFα signaling *via* NFκB gene sets (**Figure 8C**).

### scRNA-Seq Analysis Reveals the Presence of Distinct Cancer Cell Populations Expressing RBP7

To understand how the tumor cells influence the immune microenvironment, Lee et al. analyzed the transcriptome of 91,103 single cells from 23 Korean and 6 Belgian patients. Their result showed that intercellular network reconstruction supported the link between cancer cell signatures and specific matrix or immune cell populations (22). Based on the Lee et al. study results, we preliminarily understood the distribution of RBP7 in colorectal cancer. RBP7 was centrally distributed in endothelial stalk cells, endothelial tip cells, and myeloid cells (**Figures 8D, E** **)**.

### Verification of the Relationship Between RBP7 Level and 5-FU Sensitivity in Cells

**Figure 9:**
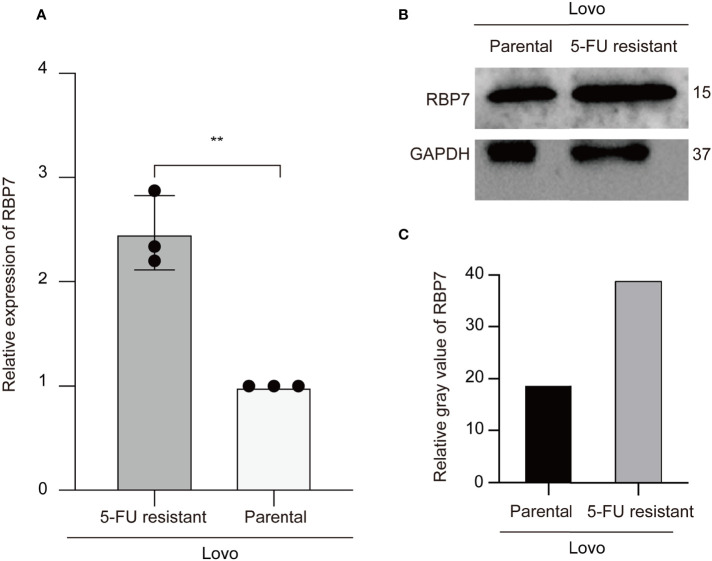
RBP7 high expressed in the 5-FU resistant Lovo cells. **(A)** The mRNA expression of RBP7 increased in the 5-FU resistant Lovo cells. **(B)** The protein expression of RBP7 increased in the 5-FU resistant Lovo cells. **(C)** The relative gray value of RBP7 for western blot analysis. **p-value < 0.001.

To evaluate whether RBP7 mediates the response of CRC cells to 5-FU treatment, we preliminarily verified in cell experiments that both transcription level and protein level of RBP7 were increased in 5-FU resistant cells (**Figure 9**), further suggesting that RBP7 might induce 5-FU resistance in CRC.

## Discussion

CRC is a malignancy with a high incidence and mortality in the world. For many years, 5-FU based chemotherapy has been used as first-line treatment for CRC patients (23). Although drug therapy is effective for most CRC patients in the initial stage, the consequential resistance may cause the poor prognosis of cancer patients. To date, 5-FU based chemotherapy remains the first-line therapy for CRC. Therefore, understanding the mechanisms of chemoresistance in CRC is essential to optimizing current therapeutic strategies.

It has been well known that TME, and especially its immune response, is essential for the regulation of tumor process and therapy responsiveness (17). Moreover, antitumor agents, including fluoropyrimidines, irinotecan, and oxaliplatin, have local and systemic immunomodulatory effects beyond their cytostatic mechanisms (24–26). Preclinical models illustrate that chemotherapy can change immune states such as, acting immune effector cells, inhibiting immunosuppressive, increasing antigenicity, immunogenicity, or susceptibility to immune attack through other mechanisms (26–29). Fluoropyrimidines optionally deplete immunosuppressive myeloid-derived suppressor cells (MDSCs) (30), and have also been correlated to a pro-tumor Th17 response (31, 32). In relation to the clinical correlation, patients undergoing neoadjuvant 5-FU/oxaliplatin showed added infiltration of CD3+ (33, 34), natural killer (NK), and CD8+ cells (35) in resected liver metastases compared with patients receiving early surgery. Overall, 5-FU based chemotherapy induced potential changes in the microenvironment and the regulation of the microenvironment may provide a new strategy for reversing the drug resistance.

We first clarified the expression of immune-related genes of 5-FU resistance in CRC. Multivariate Cox analysis suggested that HSPA8, RARB, RABEP2, ICAM2, CHGB, GALP, ICOS, RELA, and CSH2 were associated with DFS of CRC patients. CCL22, FABP7, LTBR, and RBP7 showed significance in OS of CRC patients. Moreover, we researched the differently expressed genes of the the tumor microenvironment. Based on the above 13 prognostic IRGs, we further confirmed RBP7 as a tumor immune-related microenvironment prognostic gene of 5-FU resistance. Interestingly, this prognostic gene had a good performance in predicting the prognosis of CRC patients.

RBP7 is a member of the cellular retinol-binding protein family (36) and is necessary for vitamin A stability and metabolism (37). It has been widely accepted that vitamin A, and its metabolic products, are involved in epithelial cell proliferation, differentiation, and apoptosis (38). CRBP members and retinol signaling may participate in colon cancer progression, cancer stem cell traits, tumor aggression, and EMT (39–41). RBP7 has been demonstrated as a prognostic biomarker and linked to invasion and EMT in colon cancer but the role of RBP7 in 5-FU chemotherapy resistance remains unknown.

Our study showed that RBP7 was significantly differentially expressed between 5-FU resistant patients and 5-FU sensitive patients. We found that RBP7 was lower expressed in tumor tissue than in normal but higher expressed in 5-FU resistant tumor tissues than in 5-FU sensitive tissues. This unregular expression pattern is worth further study. At this stage, we have two hypotheses: one is that RBP7 might have a dual role in tumor and the other is that RBP7 may be genetically altered when expressed in colorectal tumor tissue. This result indicated that RBP7 may be a new marker for predicting 5-FU resistance. In addition, the expression pattern of RBP7 in the the tumor microenvironment suggested that RBP7 may mediate 5-FU resistance by regulating the tumor microenvironment. As GSEA analysis results showed, KEGG pathways associated with RBP7 expression was mostly enriched in inflammation, cytokine, and chemokine signaling pathways. Single-cell RNA-seq suggested that RBP7 tended to express in microvascular cells. GSVA analysis indicated that RBP7 was related to cellular oxygen metabolism. In patients with high expression of RBP7, the gene of hypoxia signal was significantly down-regulated. Therefore, we think that RBP7 may have a positive association with intracellular oxygen transport. In addition, the results of single cell analysis suggested that RBP7 was highly expressed mainly in the vascularized system, thus confirmed the correlation between RBP7 and cellular oxygen content to some degree. In addition, GSVA results suggested that downregulation of the tumor necrosis factor pathway might induce chemotherapeutic resistance. As a proinflammatory factor, the tumor necrosis factor is closely related to the occurrence of cancer. However, its biological functions are diverse, which may promote tumor development and also may play an anti-tumor role. Some studies have shown that it could induce the disruption of tumor vasculature to achieve anti-tumor effects (42, 43). Low levels of TNFα expression could be also pro-tumorigenic (44). Based on the information above, we boldly presume that RBP7 may function on drug-resistant dormant cells and promote the cells transforming.

There are four CRBPs that can be found in humans, encoded by the *RBP1*, *RBP2*, *RBP5*, and *RBP7* genes (45, 46). A study has shown that specific subclasses of endogenous lipids interacted with CRBP2 (47) which revealed that CRBP2 might transport not only retinol but also other lipids. Other studies have shown that CRBP-III functioned as a PPARgamma target gene and played a role in lipid metabolism (48). Similarly, CRBP-I regulated adipocyte differentiation by affecting PPAR gamma activity for its a cytosolic protein specifically expressed in preadipocytes (49). Jinsoo, et al. indicated that exposure to cold led to an increased expression of RBP7 in brown adipose tissue (BAT) (50). Lipid metabolism is associated with chemotherapeutic resistance. In view of the potential role of RBP7 in lipid metabolism, further research on the relationship between RBP7 and lipid metabolism pathway may be the entry point to study its role in chemotherapy resistance.

There is no doubt that our study had some limitations. First, a prospective study should be carried out to validate the findings for this study as it was a retrospective study. Second, *in vivo* and *vitro* studies should be performed to explore reliable molecular mechanisms.

In conclusion, this study indicates that immune-related genes will hopefully be potential prognostic biomarkers to predict chemotherapy resistance for CRC. RBP7 may function as a tumor microenvironment regulator to induce 5-FU resistance, thereby affecting the prognosis of CRC patients.

## Data Availability Statement

This data can be found here: https://www.ncbi.nlm.nih.gov/geo/query/acc.cgi

https://www.immport.org/shared/genelists

https://gdc.xenahubs.net/download/TCGA-COAD.htseq_fpkm.tsv.gz

https://gdc.xenahubs.net/download/TCGA-READ.htseq_fpkm.tsv.gz. The accession number(s) can be found in the article/supplementary material.

## Author Contributions

XH: Data collection and analysis, Investigation, Visualization, Writing-Original draft. KK, WJ, QiaZ, QicZ, RM, RZ, SY, and LS: Review, Technical support. XuS, JF, and TD: Review, Technical support, Funding acquisition. XiS, TX, QW, and YM: Conceptualization, Writing-Review & Editing, Supervision, Funding acquisition. All authors contributed to the article and approved the submitted version.

## Funding

This work was financially funded by the grants National Natural Science Foundation of China (No. 82022075, to XiS; 81730108 and 81973635, to TX; 82104207, to XuS), the Science and Technology Development Fund, Macau SAR (No. 130/2017/A3, 0099/2018/A3 and 0098/2021/A2, to QW), Zhejiang Provincial Natural Science Foundation of China (No. LQ22H280001, to XuS; LQ20H160013, to TD; LQ21H160038, to JF), and Zhejiang Province Science and Technology Project of TCM (2021ZQ058, to RZ, China). Science and Technology Planning Project of Guangdong Province (No. 2020B1212030008, to QW).

## Conflict of Interest

The authors declare that the research was conducted in the absence of any commercial or financial relationships that could be construed as a potential conflict of interest.

## Publisher’s Note

All claims expressed in this article are solely those of the authors and do not necessarily represent those of their affiliated organizations, or those of the publisher, the editors and the reviewers. Any product that may be evaluated in this article, or claim that may be made by its manufacturer, is not guaranteed or endorsed by the publisher.
